# Hypoxia signature derived from tumor-associated endothelial cells predict prognosis in gastric cancer

**DOI:** 10.3389/fcell.2025.1515681

**Published:** 2025-01-20

**Authors:** Ruiheng Wang, Guijun Liu, Ke Wang, Zhanglei Pan, Zhihua Pei, Xijiao Hu

**Affiliations:** ^1^ Surgical Ward, The Second Affiliated Hospital of Heilongjiang University of Chinese Medicine, Harbin, China; ^2^ Heilongjiang University of Chinese Medicine, Harbin, China; ^3^ Department of administrative, The Fourth Affiliated Hospital of Heilongjiang University of Chinese Medicine, Harbin, China; ^4^ Endoscopy Room, First Affiliated Hospital of Jiamusi University, Jiamusi, China; ^5^ Hubei Key Laboratory of Agricultural Bioinformatics, College of Informatics, Huazhong Agricultural University, Wuhan, China; ^6^ Department of Obstetrics and Gynecology, The Second Affiliated Hospital of Heilongjiang University of Chinese Medicine, Harbin, China; ^7^ Postdoctoral Research Station of Heilongjiang Academy of Traditional Chinese Medicine, Harbin, China

**Keywords:** gastric cancer, immunotherapy, single-cell, endothelial cells, hypoxia, prognosis

## Abstract

**Background:**

A hypoxic metabolism environment in the tumors is often associated with poor prognostic events such as tumor progression and treatment resistance. In gastric cancer, the mechanism of how hypoxia metabolism affects the tumor microenvironment and immunotherapy efficacy remains to be elucidated.

**Methods:**

We used the bulk-mapping method to analyze the signatures correlated with the response of immunotherapy in the single-cell dataset. Cellular, pathway, and gene were systematically analyzed in both single-cell and bulk validation datasets.

**Results:**

The most significant cell proportion difference between the response and non-response groups was in endothelial cells, which represent the malignant cells. VWF was specifically overexpressed in endothelial cells and was the hub gene of differential genes. EPAS1 was a VWF trans-regulated gene and highly positively correlated with VWF in expression. Knockdown experiments demonstrated that siVWF reduced the expression of VWF, EPAS1, and HIF1A, as well as the synthesis of lactate and adenosine which are indicators of hypoxic metabolism. These results suggest that the overexpression of core malign endothelial genes such as VWF drives hypoxic metabolism in tumors and creates an immunosuppressive environment that reduces the efficacy of immunotherapy. The adverse prognosis of the hypoxia signature was validated in the bulk cohort and significance was further enhanced after selecting core genes and combined survival weight scoring.

**Conclusion:**

In summary, high expression of the malignant endothelial cell driver genes VWF and EPAS1 enhances hypoxic metabolism, and malignant cell-immune cell interactions suppress the immune response. Therefore, the two core genes of hypoxic metabolism might represent potential therapeutic and predicting biomarkers for immunotherapy of gastric cancer in the future.

## 1 Introduction

Immunotherapy in gastric cancer (GC) has made significant progress in recent years, and it plays an increasingly important role in the treatment of gastric cancer. Immune checkpoint inhibitors (ICIs) in immunotherapy, restore the killing ability of immune cells to tumors by inhibiting the molecular interactions such as PD-1 and PD-L1 on the surface of tumor cells or immunosuppressive cells, then recognize the antigens of tumors and kill the tumor cells subsequently (Couzin-Frankel, 2013). Although immunotherapy has made some progress in the treatment of gastric cancer, it still faces some challenges. First, although many clinical studies have suggested that gastric cancer patients with high mutation burden (Samstein et al., 2019), microsatellite instability (Asaoka et al., 2015), or Epstein-Barr virus (EBV) positive (Shahid and Prockop, 2021) are more likely to benefit from ICIs, the genetic complexity and heterogeneity of gastric cancer affect the efficacy of immunotherapy. Therefore, the efficacy of immunotherapy does not apply to all patients with gastric cancer, and individualized treatment plans need to be formulated according to the specific conditions of patients.

In addition to immune cells themselves, other components of the tumor microenvironment also have an important impact on the progression of gastric cancer and the efficacy of immunotherapy (T, 2021). Among them, malignant endothelial cells, as abnormal cells in tumor blood vessels, play an important role in the tumorigenesis and development of gastric cancer. The malignant endothelial cells may participate in tumor immune evasion and inflammatory responses by secreting cytokines and chemokines (Kim and Cho, 2022). These cells provide favorable conditions for tumor growth and spread by promoting tumor angiogenesis and participating in immune escape mechanisms (Maishi and Hida, 2017; Sobierajska et al., 2020; Fang J. et al., 2023). In addition, malignant endothelial cells have a strong association with the malignant grade, depth of invasion, and nodal involvement in gastric cancer, which has an important impact on the prognosis of patients (Shan et al., 2022).

Moreover, hypoxic metabolism in gastric cancer is also a pathophysiological process worthy of attention. Gastric cancer cells are often in a hypoxic microenvironment due to the rapid growth of tumor tissue, insufficient blood perfusion, and anemia of patients. To adapt to this environment, gastric cancer cells undergo metabolic reprogramming, which is mainly manifested as increased glycolysis (Chen et al., 2023). This metabolic alteration not only enhances the hypoxia tolerance of tumor cells but may also affect their sensitivity to immunotherapy (Tao et al., 2022; Shi et al., 2021). Therefore, an in-depth study of the mechanism of hypoxic metabolism in gastric cancer is of great significance for improving the effect of immunotherapy and improving the prognosis of patients. Hypoxic metabolism is also associated with enhanced invasion and distant metastasis of gastric cancer cells (Zhao et al., 2022). In response to angiogenic factors released by tumors, endothelial cells can create aberrant vascular networks that aid in the metastasis and dissemination of tumor cells (Infantino et al., 2021). Under hypoxia, some specific transcription factors and signaling pathways will be activated to regulate the expression of downstream target genes, leading to changes in the biological characteristics of tumor cells (Feng et al., 2023). For example, hypoxia-inducible factor (HIF) is a key transcription factor in the cellular response to hypoxia. It is involved in tumor angiogenesis, apoptosis inhibition, cell cycle, and proliferation changes by regulating the expression of a series of target genes (17). Hypoxia changes in gene expression enhance the proliferative capacity of tumor cells and inhibit apoptosis. Under hypoxic conditions, tumor cells can alter the expression of surface antigens, secrete immunosuppressive factors, and even induce apoptosis of immune cells. These changes collectively lead to the imbalance of the tumor immune microenvironment, enabling tumor cells to cleverly evade recognition and attack by the immune system (Chen et al., 2022). Overall, hypoxic metabolism in gastric cancer is a complex and important pathophysiological process that involves multiple levels of regulation and interactions.

Hypoxic metabolism and malignant endothelial cells are significant contributors to gastric cancer and have a significant influence on immunotherapy. Therefore, this study aims to investigate the role of these factors in the immune microenvironment and their influence on immunotherapy and prognosis from multiple-omics that include single-cell, transcriptomics, and genomics. This study might inspire a new potential direction for combined treatment regimens for gastric cancer.

## 2 Materials and methods

### 2.1 Data collection

Bulk RNA-seq of the Korean gastric cancer Immunotherapy dataset (Kim. chort) was downloaded from the European Nucleotide Archive and is available under accession PRJEB25780 (Kim et al., 2018). The single-cell gastric cancer expression data GSE183904 (Kumar et al., 2022) and the two bulk molecular subtyping cohorts GSE62254 (the Asian Cancer Research Group, ACRG) (Cristescu et al., 2015; Oh et al., 2018) and GSE84437 (Yoon et al., 2020) were downloaded from GEO websites. The Cancer Genome Atlas Consortium (TCGA) expression data were downloaded from Xenabrowser of UCSC which log2FPKM matrix of TCGA hub (Goldman et al., 2020), and clinical information tables and mutation files were downloaded from cbioportal (Cerami et al., 2012).

### 2.2 Single cell analysis

Bulk data of immunotherapy for gastric cancer were grouped by efficacy and mapped to GSE183904 using scissor (R package, v2.0.0) (Sun et al., 2022). Expression normalization and clustering using Seurat (R package, v4.3.0.1) (Butler et al., 2018; Hao et al., 2021) resulted in 18 clusters. scType (R package, v1.0.0) (Ianevski et al., 2022) was used for cell annotation, while only gastric cancer cells and immune cells were selected in the database and the duplicate marker gene lists were excluded. Malignant cells were inferred using inferCNV (R package, v1.16, https://github.com/broadinstitute/inferCNV), and CD8 NKT cells were selected as the reference. Cell-to-cell interactions were calculated using CellChat (R package, v1.6.1) (Jin et al., 2023).

### 2.3 Differential expression and pathway enrichment analysis

Single-cell differential analysis was performed using the FindMarkers function of the Seurat package. The genes with significant differences were defined as an absolute log2FC value greater than 0.25 and p.adjust<0.001. Marker genes of endothelial cells were calculated using COSG (R package, v0.9.0) (Dai et al., 2022), and genes with a score>0.33 were selected. Bulk RNA-seq computed the DEGs by limma (R package, v3.56.2) (Ritchie et al., 2015) and the threshold was set to the absolute log2FC value greater than 1 and p.adjust<0.05. ClusterProfiler (R package, v4.8.2) (Wu T. et al., 2021) was used for pathway and bio-function enrichment analysis. To determine the percentage of immune cells in CIBERSORT (Zeng et al., 2021), the deconvo_tme function of the IOBR (R package, v0.99.9) was used to analyze immune cells in the bulk dataset (Newman et al., 2015). The hallmark expression score uses the value of ssGSEA computed by GSVA (R package, v1.48.3) (Hänzelmann et al., 2013) to represent the expression level of the pathway.

### 2.4 Cell culture and VWF interference assay

Primary human umbilical vein endothelial cells (HUVECs) were obtained from the Cell Resource Center, Peking Union Medical College (which is part of the National Science and Technology Infrastructure, the National Biomedical Cell-Line Resource, http://cellresource.cn) on Sept.08,2023. HUVECs were cultured in endothelial basal medium supplemented with Endothelial Cell Growth Medium and 10% fetal calf serum. RNA interference experiments were conducted in HUVECs, with the expression of β-actin serving as a control. The Supplementary Materials displays the nucleotide sequences of the siRNA and the primer of the VWF, EPAS1, and HIF1A genes.

### 2.5 Culture and VWF interference assay

Lactate and adenosine, the core products of hypoxic metabolism, were detected and quantitated using an ELISA assay. The ab65330 L-Lactate Assay Kit and Human Adenosine ELISA Kit were both purchased from COIBO BIO Biotechnology Co., Ltd. (Shanghai, China) and detected the metabolite following the manufacturers’ protocols correspondingly.

### 2.6 Endo-hypoxia score

Due to the raw hypoxia ssGSEA scores computed by full gene set could only indicate prognosis in few of the dataset, and the wide range of correlations intra the hypoxia gene set suggested the redundancy. We screened the hypoxia genes that were highly correlated with VWF and EPAS, respectively (spearman correlation >0.4, and p.value < 0.05) and then took the intersection to obtain a streamlined endothelial-derived hypoxia gene set. The coefficients for endothelial-derived hypoxia scores were calculated by taking the (−1)*log10 (p-value) of the Cox proportional-hazards model test in the Kim dataset, i.e., by the following equation:

$$hypoxia\_score=\sum_{i=1}^{7}-\log10\left({PV}_{i}\right)*{\mathrm{Exp}}_{i}$$

### 2.7 Statistics

All analyses in this paper were performed under R4.3.1. Survival and Survminer packages were used for survival analysis (Therneau and Grambsch, 2000). The Kaplan-Meier curve was employed to visualize the difference in prognosis between the two groups, and the log-rank test was utilized to compare survival rates. To determine the differences in metabolite concentration and gene expression between the control and knockdown groups, t-tests were applied to the siVWF assay results. For all other pairwise comparisons, the Wilcoxon signed-rank test was used. The dependent variables were non-linearly correlated, so Spearman’s correlation coefficient was used for the correlation test. The drug target pathway enrichment analysis used the hypergeometric test. All the p.value adjust by the Benjamini and Hochberg method.

## 3 Results

### 3.1 Tumor-associated endothelial cells are the most influential tumor cells in immunotherapy

**FIGURE 1:**
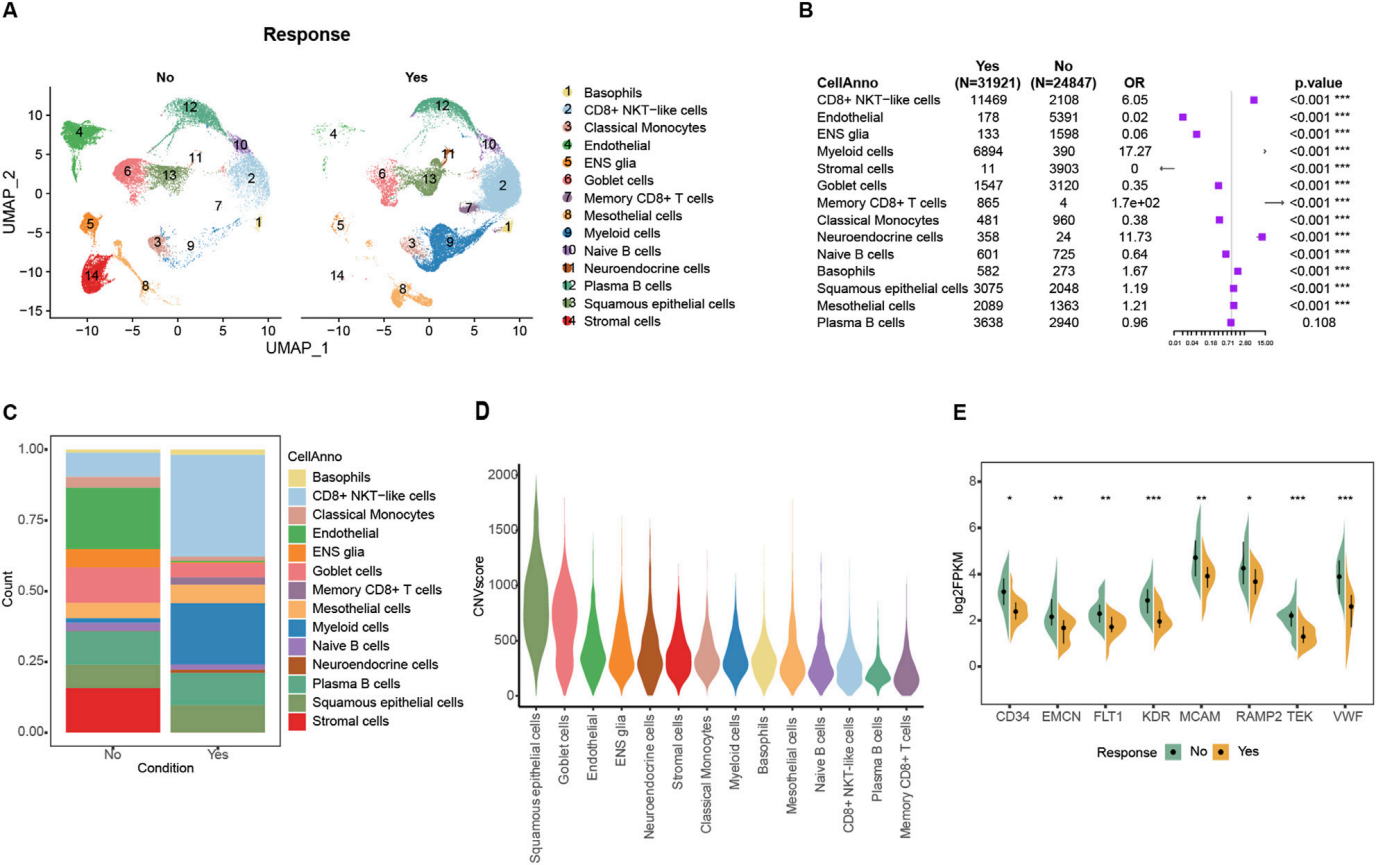
Tumor-associated endothelial cells were enriched in the non-response immunotherapy group in single-cell datasets. **(A)** Cells distribution in UMAP space of bulk mapped scRNA datasets. **(B)** Forest plot of the composition of the cells in the comparison between the mapped ICIs treatment effective and ineffective groups. **(C)** Cell proportion in mapped ICIs treatment effective and ineffective groups. **(D)** Violin plot of malignant CNV events among different cells. **(E)** Comparison of endothelial signature genes expression between response and non-response groups in Kim ICIs bulk dataset.

The Korean GC Immunotherapy Cohort (Kim) screened 45 patients with available response information and then mapped this bulk expression data to the GSE183904 single-cell data, resulting in a total of 24874 non-responder cells and 31921 responder cells. The response group was enriched in various immune cells, especially CD8^+^ NKT cells, while the most significant difference in tumor cells was endothelial cells (Figures 1A–C; Supplementary Table 1). After inferCNV computed the malign CNV events, non-immune cells result in a high degree of malignancy, and the top three are squamous epithelial cells, goblet cells, and endothelial cells (Figure 1D; Supplementary Figure 1B). Endothelial cells showed the most significant difference in composition between two groups: only 0.56% in the response group and 21.7% in the non-response group (Figure 1B). Similar results were obtained in the bulk dataset, where specific genes highly expressed in endothelial cells, such as CD34, EMCN, FLT1, KDR, MCAM, RAMP2, TEK, and VWF, were all significantly highly expressed in the non-response group (Figure 1E; Supplementary Figures 1C–J; Supplementary Table 4). The proliferation of tumor-associated endothelial cells (TAEs) in the tumor occupies more space and might related to the formation of an immunosuppressive microenvironment.

### 3.2 Hypoxic metabolism drives immunosuppression in pathway perspective

**FIGURE 2:**
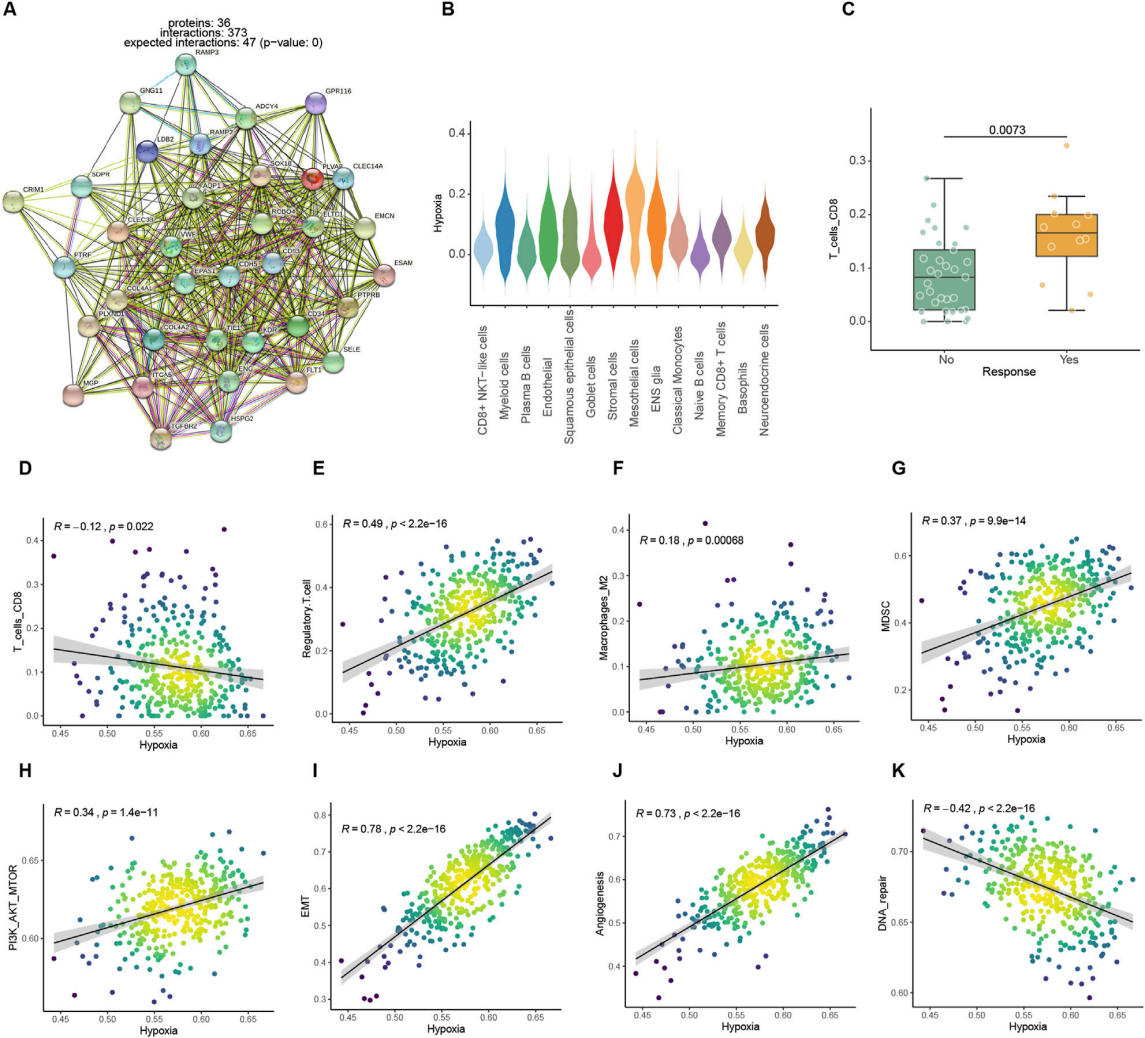
Association analysis between hypoxic metabolism and immune microenvironmental factors. **(A)** PPI networks of highly expressed endothelial cell feature genes. **(B)** Expression levels of hypoxia pathway among different cells in the ICIs mapped single-cell dataset. **(C)** Comparison of CIBERSORT CD8 T cell infiltration score between ICI treatment response and non-response in Kim bulk dataset. **(D–K)** Spearman correlation between hypoxic metabolism and immune or tumor proliferation-related factors in TCGA dataset.

Both endothelial cell-specific genes and differential genes in the Kim bulk data were enriched in the hypoxic pathway, which is one of the hallmark pathways of tumors (Figures 2A, B). Several studies reported that hypoxic metabolism was associated with immunosuppression. The responder groups in the Kim ICIs data had significantly larger numbers of CD8 T cells, which are most directly disinhibited by immunotherapy among other immune cells (Raskov et al., 2021; Ahmed et al., 2022). Additionally, there was a significant negative connection between high expression of hypoxia pathways and CD8 T lymphocyte infiltration (Figures 2C, D). In addition, the expression of an immune-suppress signature Treg (Sakaguchi et al., 2010), Macrophage M2 (Martinez et al., 2009), and myeloid-derived suppressor cell (MDSC) (Gabrilovich and Nagaraj, 2009) were also positively correlated with hypoxia metabolism (Figure 2E–J). Angiogenesis (Cao et al., 2023), cancer-associated fibroblasts (CAFs) (Qin et al., 2021), epithelial-mesenchymal transition (EMT) (Das et al., 2019), and PI3K pathways (Singh et al., 2015) related to tumor growth and invasion hypoxic metabolism were positively correlated with them (Figure 2H–J; Supplementary Figures 1A, B). While, the DNA repair pathway, which represents genomic stability, was negatively correlated with hypoxia (Figure 2K). A hypoxic metabolic environment is maintained by malignant cells and affects other cells through direct interactions or indirect metabolites, thereby further forming an immune suppression microenvironment that also promotes tumor proliferation and invasion.

### 3.3 VWF and related trans-regulated genes affect cell-to-cell interactions

**FIGURE 3:**
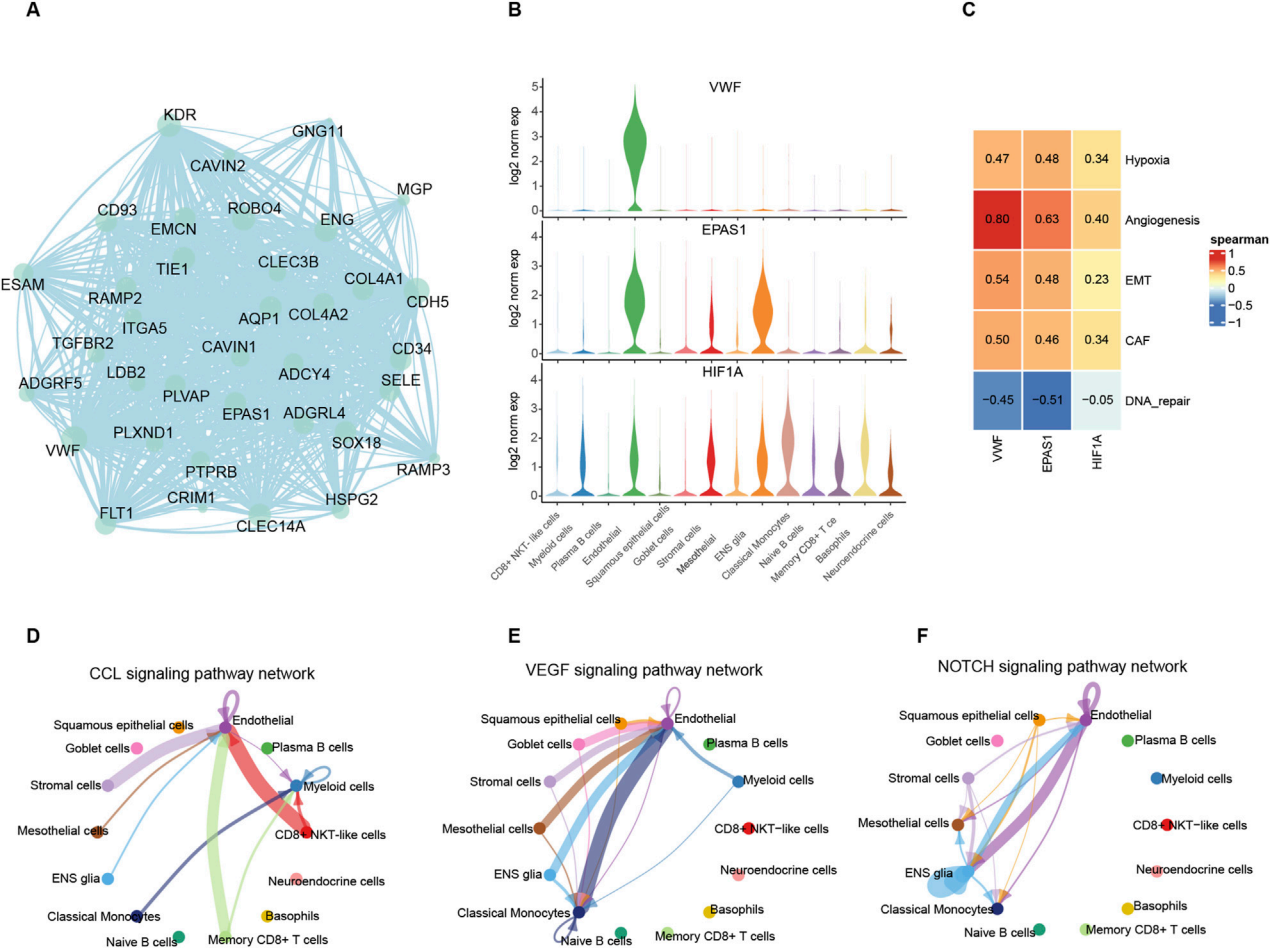
Roles of VWF in endothelial cells and communications between endothelial cells and other cells. **(A)** Weight net-graph of endothelial feature genes that VWF was the hub gene. **(B)** The expression level of the VWF, EPAS1, and HIF1A among different cells. **(C)** Heatmap of spearman correlation between three genes and factors related to hypoxia metabolism and immunity. **(D–F)** Intercellular communications which mediated by CCL, VEGF, and NOTCH pathways.

In the hypoxia pathway, VWF is one of the hub genes in the PPI network with the most protein interactions and the highest expression levels in endothelial cells compared to other signature genes (Figure 3A; Supplementary Table 5). The EPAS1, as an inducible gene under hypoxic conditions, is only expressed in endothelial cells and stroma cells. HIF1A, a hypoxia-responsive gene, is widely expressed except in epithelial cells and plasma cells. Both of these two genes positively correlated with VWF in scRNA and bulk samples (Figure 3B; Supplementary Figures 3A, B, E, F). These results suggest that VWF, as an upstream regulatory factor, affects downstream genes in other tumor cells when expressed in endothelial cells (Figure 3C). The results of cell communication indeed confirm that endothelial cells secrete cytokines and interact with other cells. While CD8 NKT cells were the immune cells that regularly interacted with endothelial cells, stroma cells were the tumor cells that were frequently associated with them (Figure 3D; Supplementary Figures 3C, D). Endothelial cells have the most cell interactions in the VEGF and NOTCH pathway which represent the proliferation process (Niu and Chen, 2010; Du et al., 2014; Brzozowa et al., 2013) (Figures 3E, F), and both pathways are involved in the negative immunomodulatory effect of MDSC cells. The prominent cell-cell interaction confirms that the most active malignant TAEs cells within the tumor are the key cells that promote the formation of an immunosuppressive microenvironment.

### 3.4 Suppressing VWF reduces hypoxic metabolism

**FIGURE 4:**
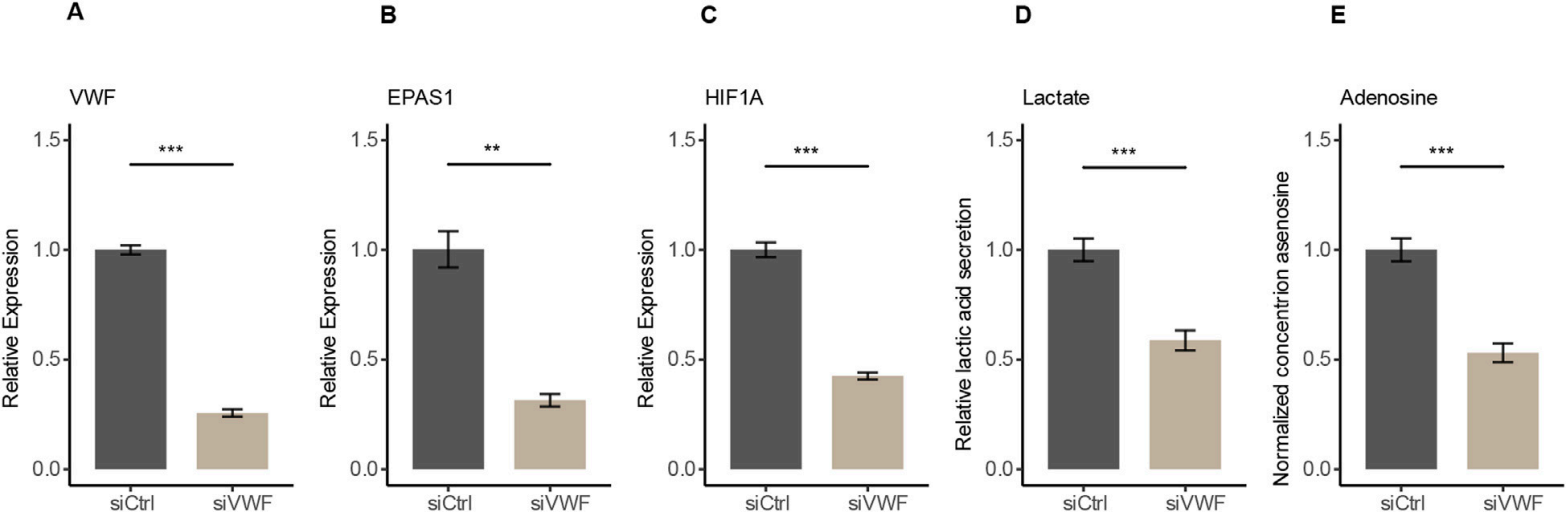
Decrease in gene expression and hypoxic metabolism after RNA interference with VWF in HUVECs. **(A–C)** After knocking down VWF, the expression of VWF, EPAS1, and HIF1A genes decreased. **(D, E)** Knockdown of VWF resulted in the reduction of the hypoxic metabolites lactate and adenosine.

To verify the regulatory role of VWF in the hypoxic pathway, we used RNA interference to knock down the expression of VWF in HUVECs. After siRNA targeting VWF (siVWF), the expressions of VWF, EPAS1, and HIF1A were all decreased (Figures 4A–C). Since EPAS1 and HIF1A are downstream response genes and positively correlated with hypoxia expression indicated that VWF has a regulatory effect on the hypoxic pathway (Supplementary Figures 4A–C). The primary features of hypoxia metabolism are elevated AMP degradation, which produces more adenosine, and enhanced glycolysis, which produces more lactic acid (Wallace et al., 2010; Pérez-Tomás and Pérez-Guillén, 2020). With the decreased expression of these three genes, the hypoxic pathway was indeed suppressed and the quantification of lactic acid and adenosine by ELISA assay was also significantly reduced in the siVWF group (Figures 4D, E). Accumulation of lactic acid can inhibit the proliferation and activation of T cells, B cells, and macrophages, while promoting the differentiation of Treg cells, further suppressing the immune response (Fischer et al., 2007; Gu et al., 2022; Antonioli et al., 2021). Adenosine mainly suppresses T cell responses through the A2AR-cAMP/PKA pathway, interfering with TCR activation and downstream signals of costimulatory molecules, resulting in reduced cytokine secretion (Haskó and Cronstein, 2004; Sorrentino et al., 2019). The siRNA assay verified that VWF promotes hypoxic metabolism and might involve in the formation of an immunosuppressive tumor microenvironment.

### 3.5 Relationship between hypoxic metabolism and tumor genome

**FIGURE 5:**
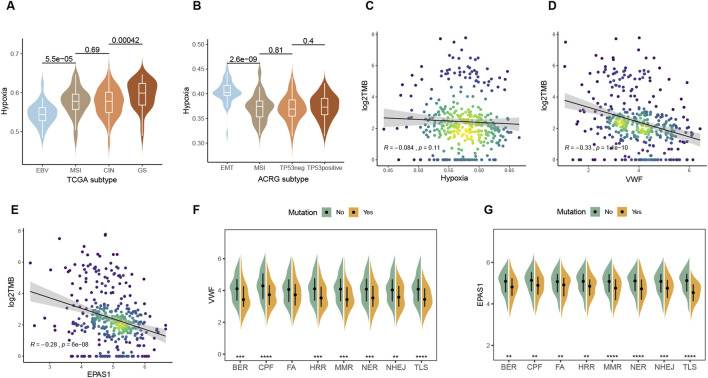
Interactions between hypoxia and the genomic factors. **(A, B)** Comparison of hypoxic metabolism among different molecular subtypes of TCGA and ACRG gastric cancer. **(C–E)** Spearman correlation between TMB and expression levels of hypoxia, VWF, and EPAS1. **(F, G)** Effect of mutations in the eight major pathways of DDR on the expression of VWF and EPAS1.

Genomic characteristics are closely related to the degree of malignancy, treatment efficacy, recurrence, and other clinical factors. Immunotherapy is more likely to benefit EBV-positive and MSI patients in the TCGA and ACRG molecular subtypes, while both of the two subtypes had low levels of hypoxic metabolism (Figures 5A, B; Supplementary Figures 1A–D). The genome-stable subtype with fewer mutations is difficult to select for drug therapy, while the EMT type is highly proliferative, prone to relapse, and has a poor prognosis. Among all the subtypes, these two have the highest hypoxia metabolism. Hypoxia metabolism and (Tumor mutation burden) TMB have a modest correlation, while VWF and EPAS1 are negatively correlated with TMB significantly (Figures 5C–E). Since TMB is a positive factor for immunotherapy, the above results indicate that high expression of VWF or abundant malignant endothelial cells may inhibit the effectiveness of immunotherapy and result in a poor prognosis. Consistent with TMB, DNA damage repair (DDR) pathway defect samples had lower hypoxic metabolism within the eight core DDR pathways (Wang et al., 2018) (Figures 5F, G). Overall tumor patients with high genomic stability prefer to be accompanied by higher hypoxic metabolism, both of which are adverse factors for immunotherapy.

### 3.6 The endo-derived hypoxia signature can guide the prognosis of gastric cancer

**FIGURE 6:**
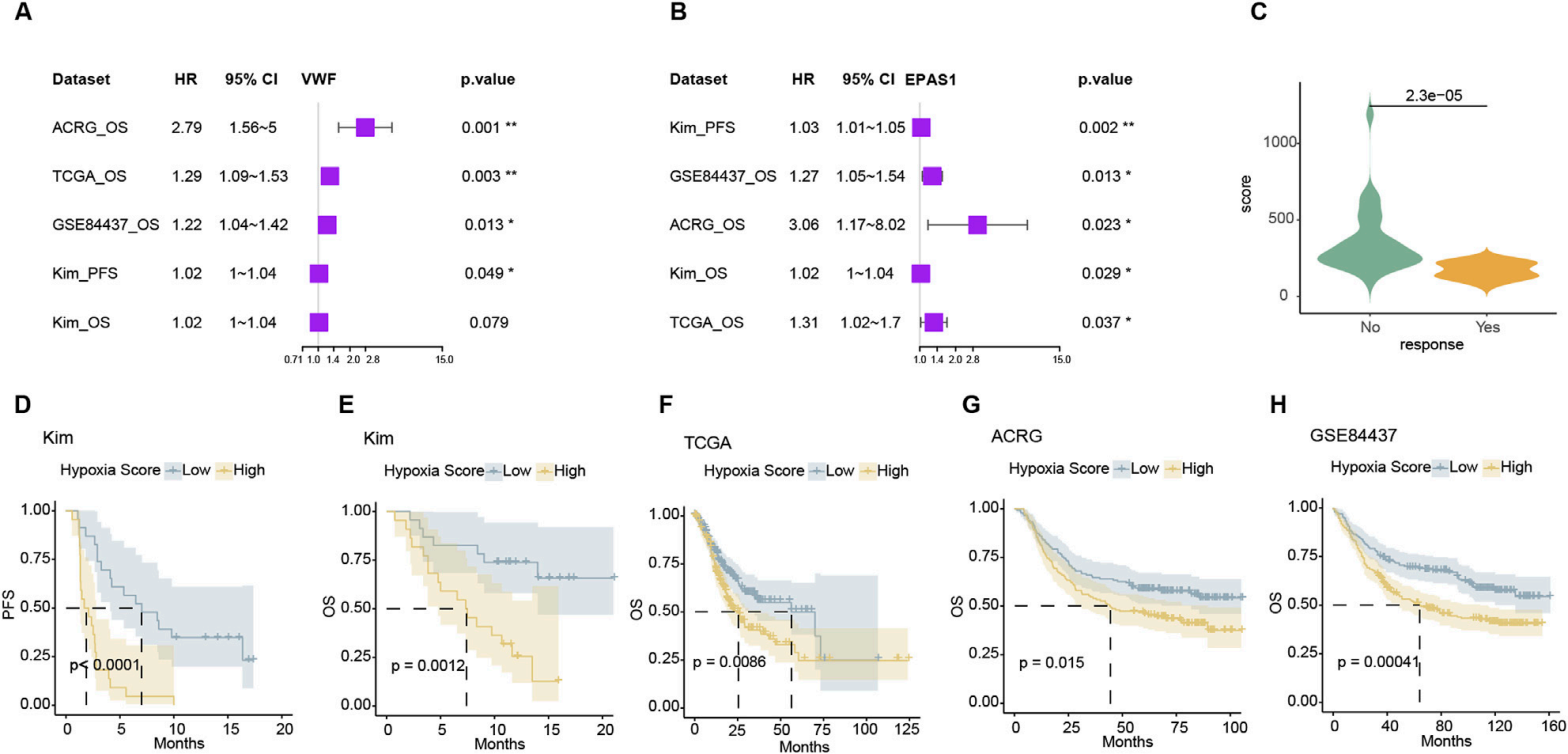
The expression signature of hypoxia can distinguish the prognosis of different data sets **(A, B)**. Forest plot of VWF and EPAS1 in four datasets that high expression with bad prognosis. **(C)** The weighted hypoxia expression scores were significantly lower in the non-response group in the Kim cohort. **(D–H)** Kaplan-Meier survival curves of the weighted hypoxia expression scores in Kim and TCGA, ACRG, and GSE84437 datasets.

For hypoxic metabolism genes, we screened those that were highly expressed in endothelial cells and showed significant differences in treatment response and survival in the Kim immunotherapy cohort. Representative genes include VWF and EPAS1 that allow for prognosis differentiation in various gastric cancer treatment cohorts as well as response and survival discrimination in immunotherapy (Figures 6A, B). The ssGSEA score of the hallmark hypoxia pathway represented the expression level of hypoxia. The significance of hypoxia survival tests can only be observed in TCGA, ACRG, and GSE84457. Indicating the variable importance of genes within the hypoxic pathway was disunity and redundant (Supplementary Figure 6A). The weak correlation between the expression levels of some hypoxic metabolism genes suggests that key genes can be selected to represent the hypoxic metabolism status of the tumor. After further eliminating genes which weakly associated with VWF and EPAS, we obtained lead hypoxic genes. For these hypoxic metabolism genes, we calculated this endo-derived hypoxia signature score using the Cox-hazard survival test −10*logPV as the weight from the Kim cohort (Supplementary Figure 6B; Supplementary Table 8). This score was significant higher in response group than non-response group (Figure 6C) and enable stratified survival outcomes across four cohorts (Figure 6D–H). The resistance mechanisms of immunotherapy and chemotherapy were similar in mechanism: hypoxia metabolism promotes proliferation and suppression of immunity, which can easily predispose to drug resistance. Consequently, the endothelial hypoxic metabolism signature has potential clinical application value in the future.

### 3.7 High endo-derived hypoxia groups might be suited for specific target therapy combined with ICIs

**FIGURE 7:**
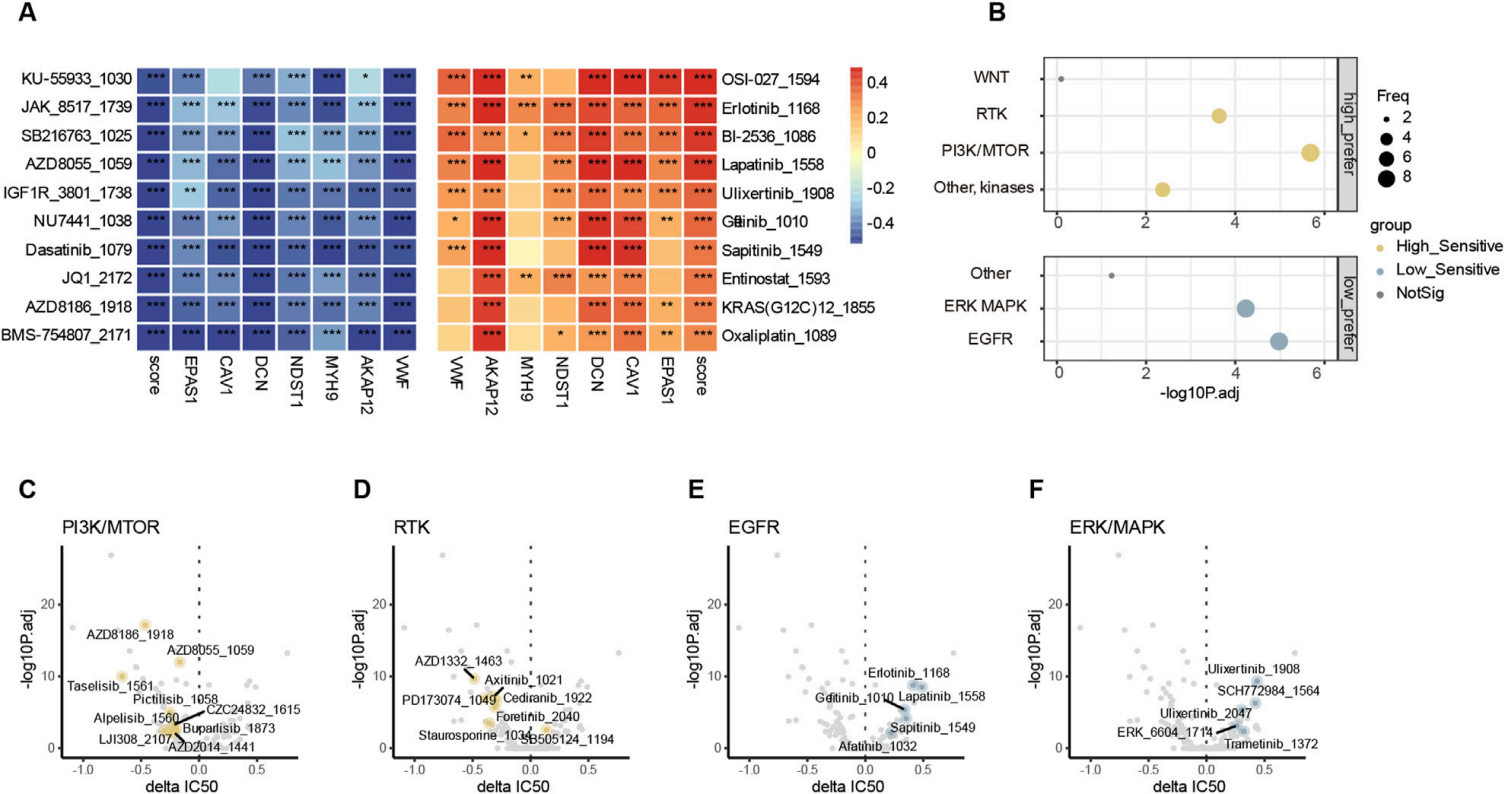
Drug sensitivity analysis **(A)**. Top 10 endo-derived hypoxia score positively (red) and negatively (blue) drug sensitivity correlated drugs. “*”, “**”, “***” represent p.adjust<0.05, 0.01, 0.001 correspondingly. **(B)** comparison of pathway enrichment target by sensitive drugs between high and low endo-derived hypoxia score groups. Delta IC50(high-low)>0 and p.adjust<0.01 defined the low prefer, while Delta IC50(high-low)< 0 and p.adjust<0.01 defined the high prefer. **(C–F)** *Volcano* plots showing differences in IC50 values between endo-derived hypoxis score high and low groups. Drugs with significant preference in four pathways are highlighted and labeled.

Since patients with low endo-derived hypoxia scores prefer to have a better prognosis than the high group, the treatment regimen applicable to patients with high scores becomes an urgent issue to be addressed. We used oncoPredict (Maeser et al., 2021) for drug sensitivity analysis in bulk RNA-seq, where the GDSC V2 training set includes transcriptomes of more than 1,000 cancer cell lines and corresponding IC50 results for 198 cancer drugs (Yang et al., 2013). The drug-sensitive analysis revealed that the sensitive drugs for patients with high and low scores were enriched in different pathways. The score negatively correlated drugs and high-score group sensitive drugs were enriched in pathways PI3K and RTK (Figures 7A–D; Supplemental Tables S11, S12, S14). The score positively correlated drugs and low-score group sensitive drugs were enriched in pathways ERK/MAPK and EGFR (Figures 7A, B, D, E, F; Supplemental Tables S11, S12, S14). Patients with high endo-derived hypoxia scores are prone to drug resistance or ineffective in chemotherapy and immunotherapy. The group with high scores might be suitable for targeted drug therapy of corresponding mutated genes in the PI3K or RTK pathways.

Furthermore, both the most significant negatively correlated and most sensitive drug in high group were BMS-754807_2171 which is an IGF1R inhibitor (Supplemental Tables S11, S13). IGF1R can activate both PI3K and RTK pathways to promote tumor proliferation and invasion (Guan et al., 2023). In the high endo-derived hypoxia score group, it might be considered to combine IGF1R inhibitor with other treatments such as cis-platinum and ICIs to improve the prognosis.

## 4 Discussion

Tumor-associated endothelial cells are an important component of the tumor microenvironment and are involved in multiple processes such as tumor angiogenesis, growth, and metastasis. During tumorigenesis, endothelial cells change their morphology, biological functions, and gene expression patterns. The malignant endothelial cells play a critical role in the tumor microenvironment by influencing angiogenesis, the immune system, and metastasis, all of which contribute to the growth and promotion of tumors. Further research on malignant endothelial cells could help reveal the mechanisms of tumor progression and drug resistance, and provide potential targets for the development of new tumor treatment methods. Hypoxia metabolism in tumors is an extremely complex and crucial biological process that primarily occurs in hypoxic regions within the tumor, contrasting sharply with the metabolic pattern in normoxic areas. This process has profound impacts on tumor growth, progression, and therapeutic response (Paredes et al., 2021). Hypoxia contribute to the imbalance of the tumor immune microenvironment, enabling tumor cells to cleverly evade recognition and attack by the immune system (Chen et al., 2022). This immune evasion ability allows tumor cells to survive and proliferate continuously within the immune microenvironment.

Hypoxia metabolism also interacts with other tumor characteristics, compounding the complexity of tumor treatment. For instance, it promotes the infiltration and metastasis of tumor cells, making it easier for them to invade surrounding tissues and organs. Simultaneously, hypoxia metabolism reduces the sensitivity of tumor cells to radiotherapy, chemotherapy, and immunotherapy, further complicating treatment (Codony and Tavassoli, 2021; Muz et al., 2015). VWF is both a characteristic gene of endothelial cells and an upstream gene of hypoxia metabolism, playing a crucial role in tumor growth, metastasis, and angiogenesis. Firstly, VWF is a key regulatory factor in primary hemostasis and a potential cause of various vascular diseases. Multiple studies have shown that VWF promotes adhesion among tumor cells by mediating interactions with surface molecules, enhancing their motility, invasiveness, and ability to degrade the extracellular matrix, ultimately facilitating tumor metastasis (Patmore et al., 2020; Yang et al., 2015). Secondly, knockout experiments have demonstrated that VWF positively regulates the hypoxia metabolism process. VWF in malignant cells establishes a hypoxic metabolic environment and promotes the formation of an immunosuppressive microenvironment.

Currently, most IGF1R inhibitor drugs are in clinical trials. In other cancers, it has been reported that targeting IGF1R signaling can enhance the sensitivity of cisplatin in oesophageal squamous cell carcinoma (OSCC). IGF1R was upregulated in OSCC under hypoxic conditions. Clinically, the increased expression of IGF1R was associated with high tumor grade and poor prognosis in OSCC patients, and its inhibitor linsitinib had synergistic effects with cisplatin treatment *in vitro* and *in vivo*. Under hypoxic conditions, the IGF1R pathway promoted the expression of metabolic enzymes ASS1 and PYCR1 through the transcriptional activity of c-MYC. The increased expression of ASS1 promotes arginine metabolism for biosynthesis, while PYCR1 activates proline metabolism to maintain redox balance, thereby reducing the sensitivity of OSCC to cisplatin under hypoxic conditions. Inhibition of IGF1R enabled to enhance the sensitivity of OSCC to cisplatin both *in vitro* and *in vivo* (Fang K. et al., 2023). In the murine model of subcutaneous transplantation tumors, picropodophyllin (PPP, an IGFR inhibitor) has been found to improve the therapeutic efficacy of chemoimmunotherapy with a combination of oxaliplatin and blocker (Wu Q. et al., 2021). Therefore, in the future, the higher endo-derived hypoxia score patients with drug-resistant populations might consider the use of IGF1R inhibitors combined with chemotherapy or immunotherapy for the treatment. Also, more in-depth biological experiments and clinical studies with real GC cohorts are urgently needed.

In summary, a large number of malignant TAEs cells highly express hypoxia metabolism-related genes, promoting tumor proliferation and invasion through various mechanisms, affecting the balance of the immune microenvironment, and interacting with other tumor characteristics, all of which increase the difficulty of treatment. Therefore, intervention strategies targeting hypoxia metabolism are of significant importance in tumor treatment, potentially providing new ideas and methods for reshaping the tumor immune microenvironment and improving treatment outcomes.

## 5 Conclusion

Malignant endothelial cells highly express VWF and regulate downstream hypoxic response genes. At the same time, malignant endothelial cells interact directly with other cells and indirectly through metabolites, establishing a microenvironment within the tumor that is conducive to proliferation, invasion, and immune suppression. The screened and de-redundant hypoxic metabolism signature can guide the prognosis of various treatment approaches. The findings of this study provide valuable insights into the development of precision medicine in the treatment of gastric cancer.

## Data Availability

The original contributions presented in the study are included in the article/Supplementary Material, further inquiries can be directed to the corresponding author.

## Abbreviations

GC, Gastric cancer; ICIs, Immune checkpoint inhibitors; TAE, tumor-associated endothelial cells; TCGA, The Cancer Genome Atlas; ACRG, Asian Cancer Research Group; TMB, Tumor mutation burden; CNV, Copy number variation; MSI, Microsatellite instability; EMT, Epithelial-mesenchymal transition; CAF, Cancer associate fibroblast; HUVEC, human umbilical vein endothelial cells; PPI, protein-protein interations; DDR, DNA damage repair; HRR, Homologous recombination repair; MMR, Mismatch repair; BER, Base excision repair; NER, Nucleotide excision repair; NHEJ, Nonho-mologous End-joining; CPF, Check Point Factors; FA, Fanconi anemia; TLS, Translesion Synthesis.
