# Supplementary figures and images for: Hypoxia signature derived from tumor-associated endothelial cells predict prognosis in gastric cancer
Source: Front Cell Dev Biol. 2025 Jan 20;13:1515681. doi: 10.3389/fcell.2025.1515681 (PMC11788339; doi:10.3389/fcell.2025.1515681)

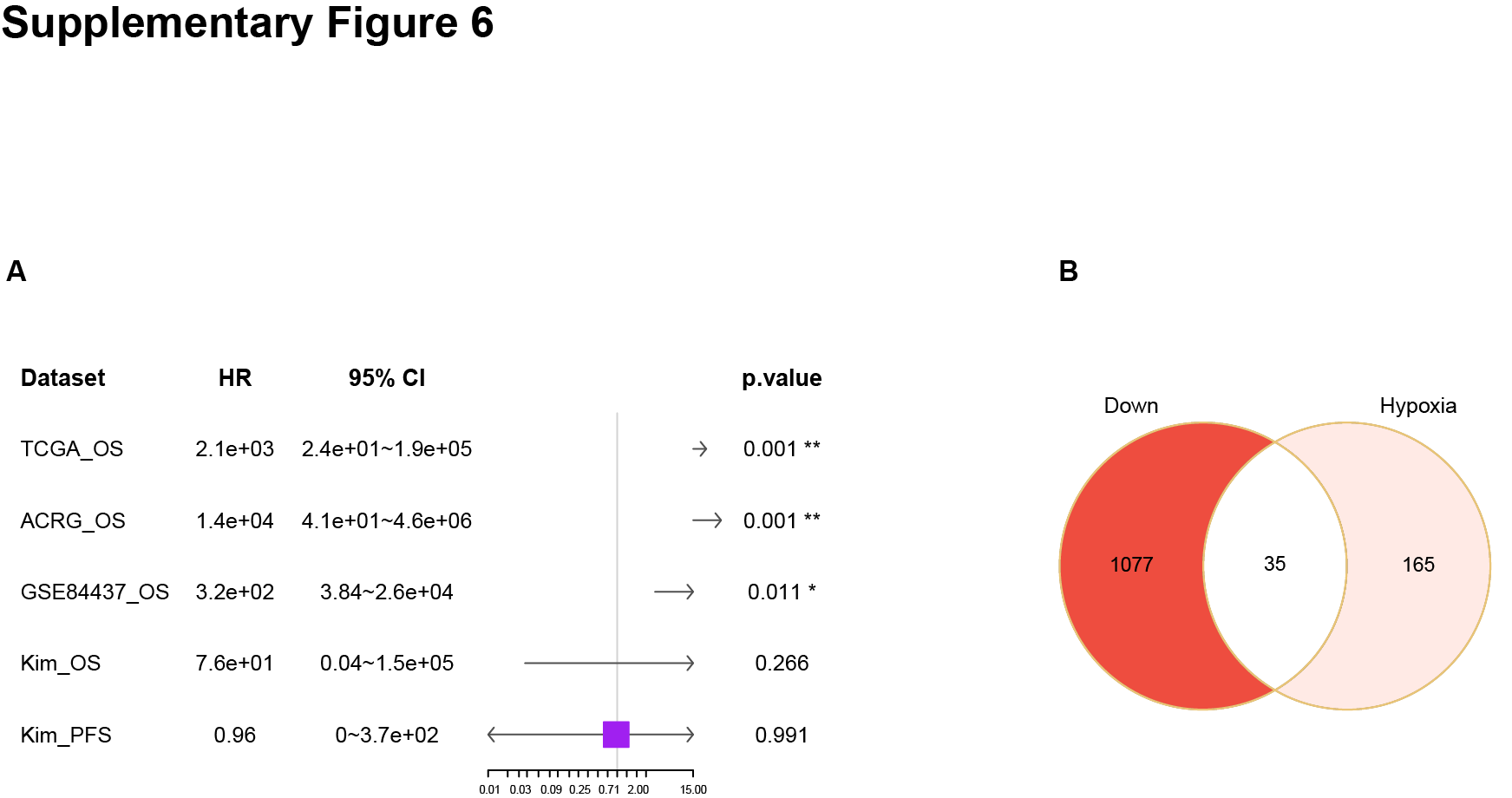

Supplement: Supplementary file 1 [file Image6.tif]

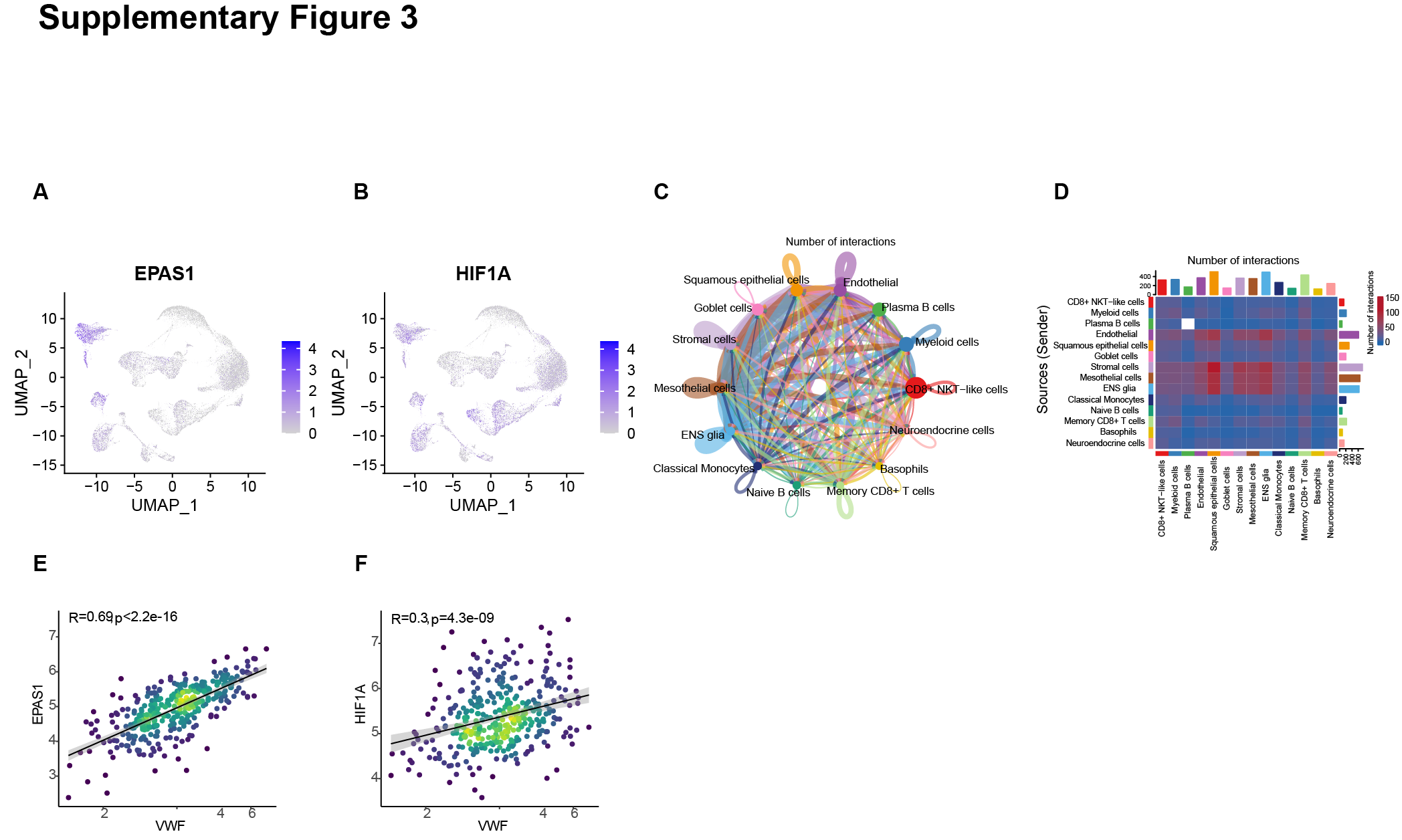

Supplement: Supplementary file 2 [file Image3.tif]

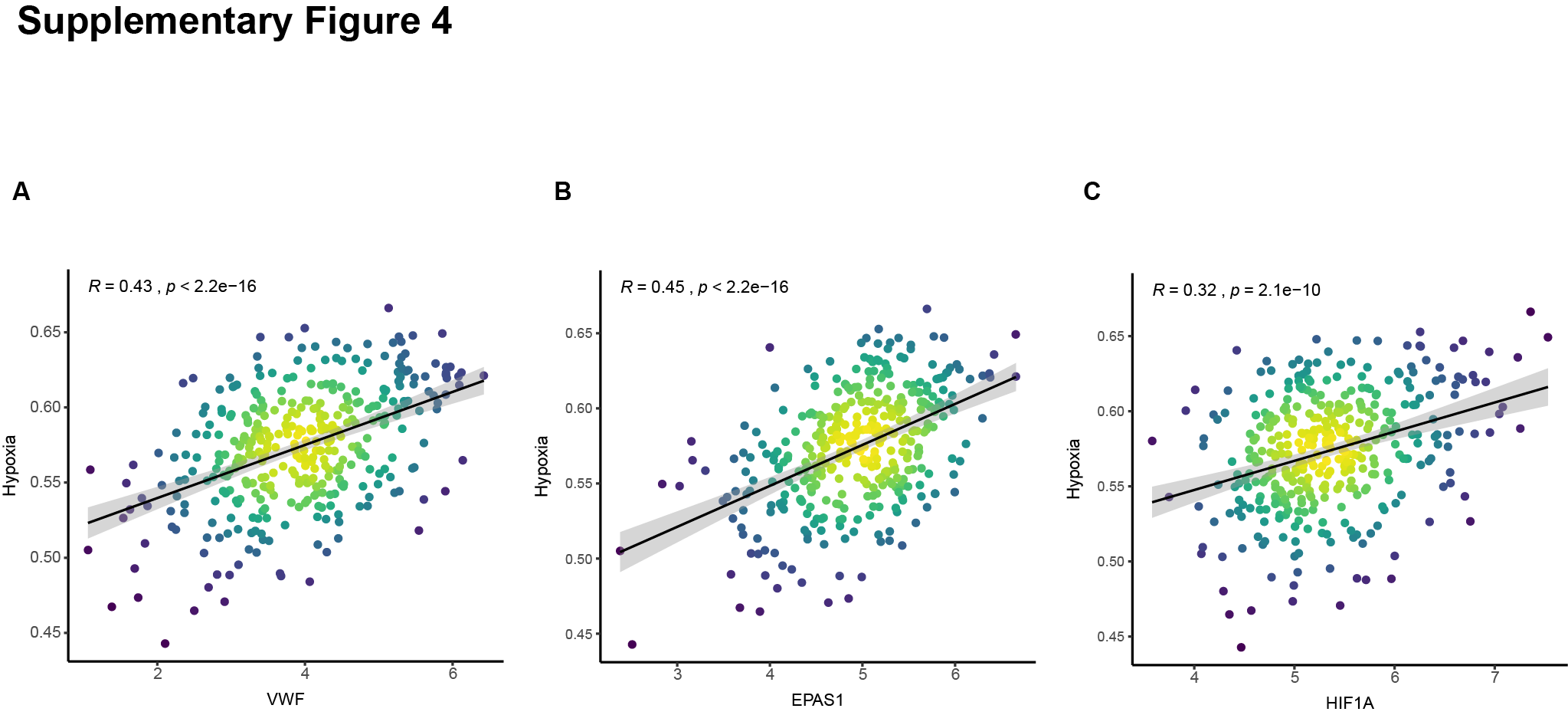

Supplement: Supplementary file 3 [file Image4.tif]

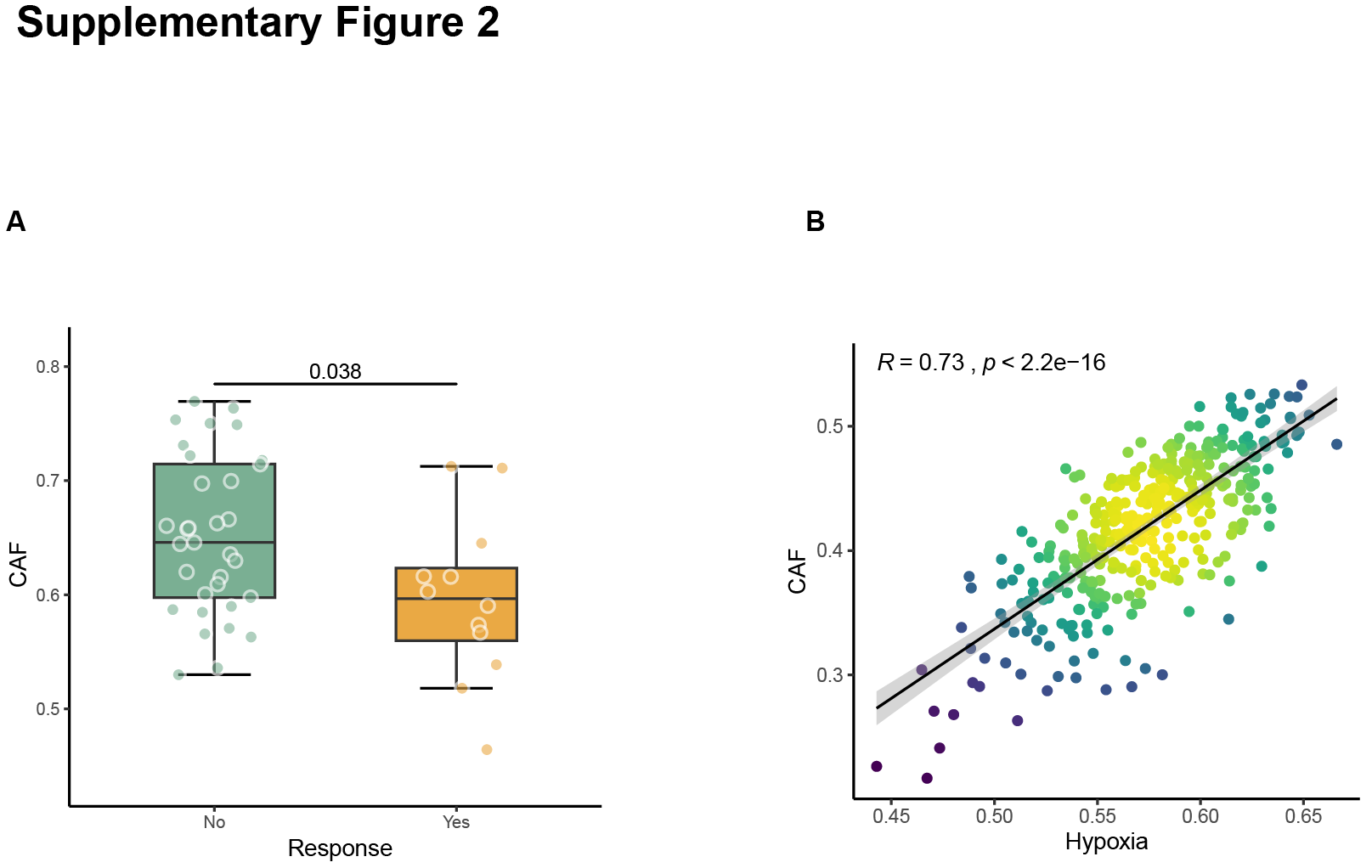

Supplement: Supplementary file 4 [file Image2.tif]

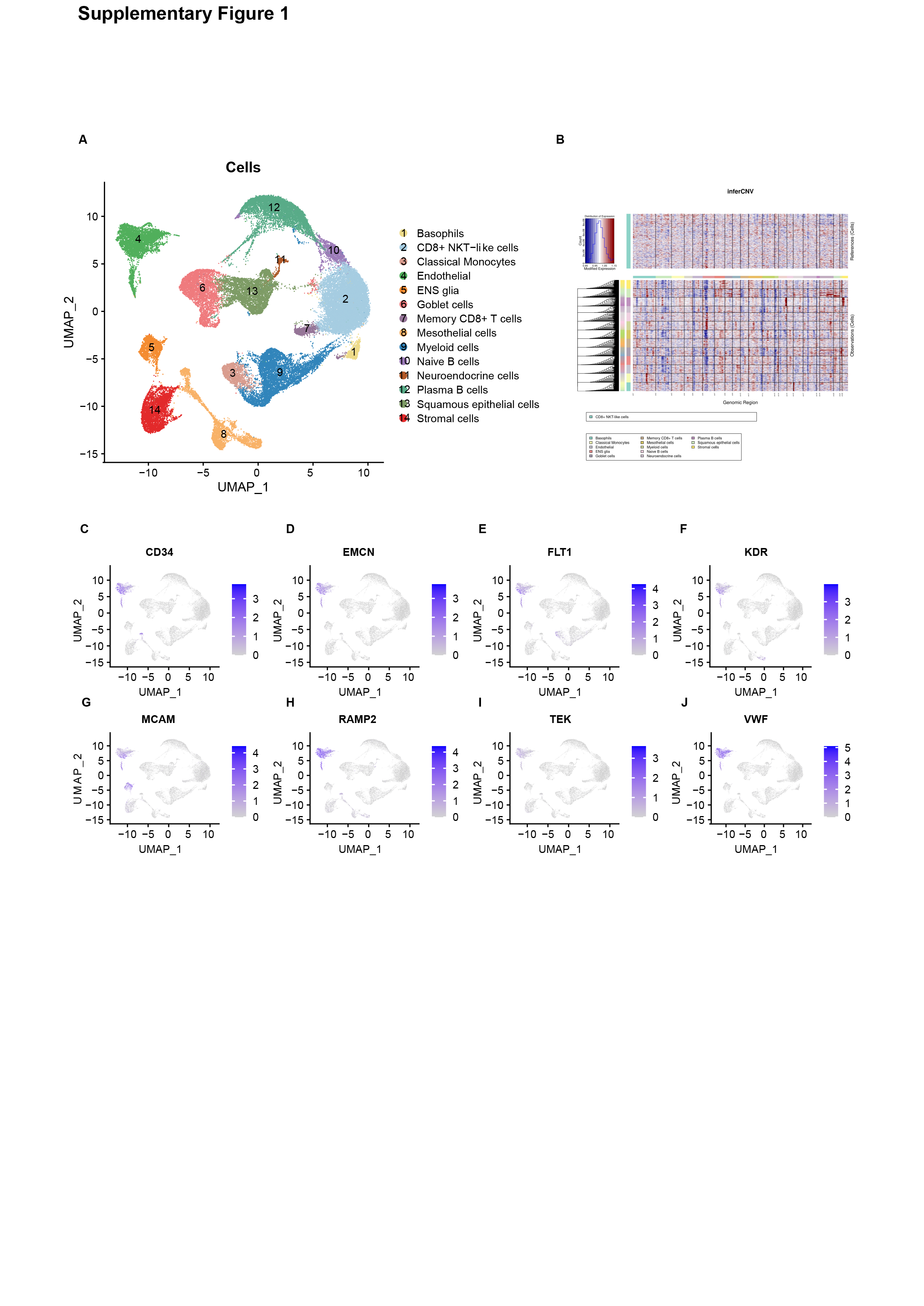

Supplement: Supplementary file 5 [file Image1.tif]

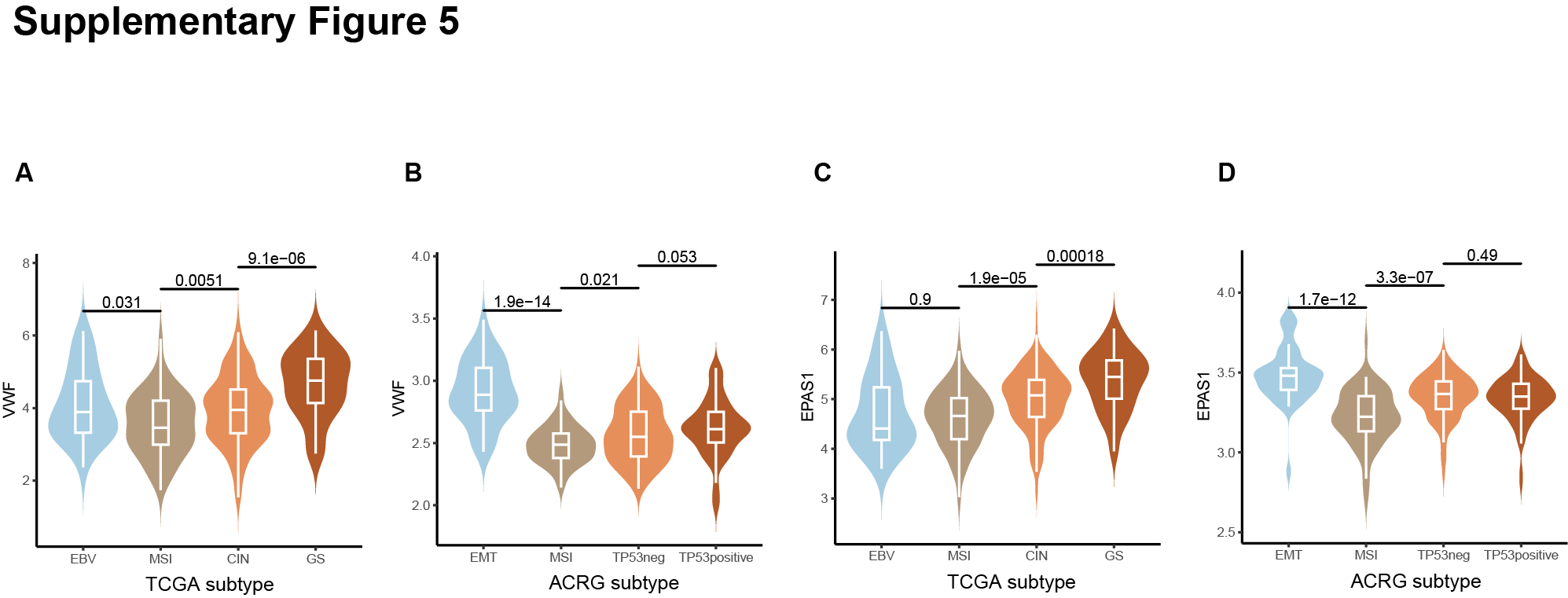

Supplement: Supplementary file 6 [file Image5.tif]
