# Supplementary material for: Hypoxia signature derived from tumor-associated endothelial cells predict prognosis in gastric cancer
Source: Front Cell Dev Biol. 2025 Jan 20;13:1515681. doi: 10.3389/fcell.2025.1515681 (PMC11788339; doi:10.3389/fcell.2025.1515681)
Supplement: Supplementary file 7 [file DataSheet1.docx]

Supplementary Material

# Supplementary Data

Primer Sequence

VWF-F CCCTGTCTCATCGCAGCAAA

VWF-R GGCACGTTTTGGTACACTCG

β-actin-F CATgTACgTTgCTATCCAggC

β-actin-R CTCCTTAATgTCACgCACgAT

EPAS1-F CGCGCACCTCGGACC

EPAS1-R CTCTCCGAGCTACTCCTTTTCTT

HIF1A-F GAGAGGTTGAGGGACGGAGA

HIF1A-R CGACGTTCAGAACTTATCCTACCAT

siRNA sequence

UAGCUGAAUGGUGCAAGCCGUdTdT

# Supplementary Tables

Table S1. Sample cluster and cell annotation of scRNA

Table S2. Overall survival test for genes expression in Kim cohort

Table S3. Progress-free survival test for genes expression in Kim cohort

Table S4. DEGs in ICBs response comparision of Kim chort

Table S5. DEGs in mapped Response group of scRNA GSE183904

Table S6. Endothelial cells marker gene scored by COSG

Table S7. Hypoxia genes correlation with VWF

Table S8. Hypoxia genes correlation with EPAS1

Table S9. Endo-derived hypoxia score weights

Table S10. RNAi assay

Table S11. Spearman correlation between endo-derive hypoxia score and drug IC50

Table S12. P.value of spearman correlation test between endo-derive hypoxia score and drug IC51

Table S13. Drug sensitivity comparison between high and low endo-derived hypoxia score groups

Table S14. Hypergeometric test of drug target enriched Pathway.

# Supplementary Figures

Supplementary Figure 1. Expression patterns of endothelial cells

A. Landscape of mapped total cells in UMAP space. B. heatmap of CNV events in different cells. C~J. Expression patterns of endothelial cells feature genes.

Supplementary Figure 2. The relationship between tumor-associated fibrocytes and immunotherapy and hypoxic metabolism.

A. Comparison of CAF ssGSEA score between response and non-response groups in the Kim ICIs cohort. B. Spearman correlation of CAF and hypoxic metabolism.

Supplementary Figure 3. Interactions between endothelial cells and other cells.

A~B. Expression pattern of EPAS1 and HIF1A in UMAP space. C. Overall cell-to-cell interactions network. D. Heatmap of the number of cell-to-cell interaction events.

Supplementary Figure 4. Correlation between hypoxia metabolism and signature genes.

A~C. Spearman correlation between hypoxia and expression level of VWF, EPAS1, and HIF1A genes.

Supplementary Figure 5. Differential expression of signature genes among different molecular subtypes.

A~B. VWF expression among four molecular subtypes of TCGA and ACRG. C~D. EPAS1 expression among four molecular subtypes of TCGA and ACRG.

Supplementary Figure 6. The whole gene set of hypoxic metabolic pathways has redundant and incongruous genes.

A. Prognostic role of hypoxia score calculated by ssGSEA which full hypoxia gene list included. B. Venn diagram of the intersection of genes between down expression in mapped response group and hypoxia feature genes.
